# Factors associated with low fruit and vegetable consumption among people with severe mental ill health

**DOI:** 10.1007/s00127-023-02514-z

**Published:** 2023-06-14

**Authors:** Ben Lorimer, Gemma Traviss-Turner, Andrew Hill, Sarah Baker, Simon Gilbody, Emily Peckham

**Affiliations:** 1 Department of Health Sciences, University of York, York, UK; 2 School of Medicine, University of Leeds, Leeds, UK; 3 York St John University, York, UK; 4 York and Scarborough Teaching Hospitals NHS Foundation Trust, York, UK; 5 Hull York Medical School, York, UK

**Keywords:** Schizophrenia, Psychosis, Bipolar affective disorders, Severe mental ill health, Health risk behaviours, Poor diet

## Abstract

Severe mental ill health (SMI) is associated with excess mortality, and poor diet is one associated modifiable risk factor. This study investigated factors associated with low consumption of fruit and vegetables among people with SMI (*N* = 9914). A total of 8.4% of participants ate no portions per day, while only 15% ate 5 + portions. Individuals who never consumed fruit and vegetables or ate < 5 portions per day were more likely to be male, younger than 65, unemployed, experience poorer general health, or perceive health as unimportant. Poor diet is common among people with SMI and tailored dietary improvement interventions are required.

**Supplementary Information:**

The online version contains supplementary material available at 10.1007/s00127-023-02514-z.

## Introduction

People with severe mental ill health (SMI) have an estimated life expectancy that is 8–15 years shorter than people in the general population [1, 2]. Furthermore, this mortality gap appears to be gradually increasing over time [3]. Preventable physical diseases (e.g., cardiovascular disease) and modifiable risk factors have been demonstrated to contribute significantly to this health inequality, with one such factor being poor diet [4, 5]. For example, a 2014 survey of 1286 adults with psychosis estimated that 74% ate less than four servings of fruit and vegetables daily, and that poor diet was frequently accompanied by other unhealthy behaviours (e.g., smoking) [6]. More recently, the Closing the Gap (CtG) Health Study, which surveyed 9914 adults with schizophrenia-spectrum disorders or bipolar disorder, estimated that 85% ate less than five portions of fruits and vegetables daily [7]. Both statistics indicate that people with SMI are frequently not meeting current guidelines for fruit and vegetable consumption (i.e., at least five portions daily) [8], and that they meet such guidelines to a lesser extent than the general population (15% compared to 28%) [9]. This limited consumption may contribute to the physical health inequalities discussed previously, especially considering that fruit and vegetable consumption has been demonstrated to provide substantial benefits to human health, including reduced risk of cardiovascular disease [10]. Improving our understanding of what factors are associated with poor diet among people with SMI may enable interventions to be developed and tailored for this group. The study objectives were therefore to utilise data collected in The CtG Health Study to investigate factors associated with: (1) never eating fruit and vegetables; and (2) low consumption of fruit and vegetables (vs meeting current guidelines).

## Method

This study extends previous descriptive analyses of data gathered from The CtG Health Study, a cross-sectional survey study conducted between April 2016 and March 2020 that examined overall health and prevalence of health risk behaviours among people with SMI. Full details of the study have been reported elsewhere [7]. To summarise, adults (18 +) with a documented diagnosis of schizophrenia, bipolar disorder, or associated disorder (corresponding to ICD-10 [F20–29 or F30–31] or DSM-IV [295.x, 296.x, or 297.x] criteria) were invited to complete a self-report questionnaire. Participants were recruited from 314 primary care sites and 23 secondary care sites across England. A total of *N* = 9,914 participants completed the questionnaire, with *n* = 3,084 being recruited from primary care and *n* = 6,830 from secondary care. The mean age of the sample was 48.2 (*SD* = 14.8), with 55.4% being male, 85.2% being of white ethnicity, and 68.4% being overweight or having obesity. Table 1 describes the demographic and health information of the sample. All study procedures were approved by West Midlands Research Ethics Committee (ref: 15/WM/0444), and all participants provided written consent.

**Table 1:** Demographic and health information of the Sample (N = 9914)

Variable	*N* (%^a^) / M (SD)	Missing *N*
**Socio-demographic information**		
Gender, *n* (%)		180^b^
Male	5388 (55.4)	
Female	4289 (44.1)	
Transgender	57 (0.01)	
Age, *M* (*SD*)	48.2 (14.8)	203
Ethnicity, *n* (%)		116
White	8350 (85.2)	
Black	434 (4.4)	
Mixed	333 (3.4)	
South Asian	426 (4.3)	
Other Asian	96 (1.0)	
Other	159 (1.6)	
Employment, *n* (%)		221
In paid employment	1687 (17.4)	
Not in paid employment	8006 (82.6)	
Index of multiple deprivation, *M* (*SD*)	4.7 (2.8)	2493
**Health information**		
Body mass index, *M* (*SD*)	28.9 (6.9)	1300
Body mass index category, *n* (%)		1300
Underweight	184 (2.1)	
Healthy weight	2544 (29.5)	
Overweight	2660 (30.9)	
Having obesity	3226 (37.5)	
Importance of maintaining a healthy lifestyle, *n* (%)		78
A top priority	4475 (45.5)	
Moderately important	3839 (39.0)	
Not worried about it	1522 (15.5)	

^a^Percentages calculated using only those cases with full data (i.e., excluding missing)

^b^Total includes participants who responded “Prefer Not to Say”

The questionnaire included questions related to demographics, general health, and engagement in various health risk behaviours (Supplementary Materials A). One investigated behaviour was the consumption of fruit and vegetables, with this being assessed through the following question: “In general, how many portions of fruit and vegetables do you eat per day?”. Response options included ‘I don’t eat fruit and vegetables’, ‘one’, ‘two’, ‘three’, ‘four’, and ‘five or more’. Participants were also asked to rate their overall general health in the last year (‘excellent’, ‘good’, ‘moderate’, ‘poor’, or ‘very poor’), and how important maintaining a healthy lifestyle was to them (‘a top priority’, ‘moderately important’, or ‘I don’t worry about it’).

Two binary logistic regression models were conducted using a Bonferroni-adjusted alpha value of 0.025 (0.05/2). For one model, the dependent variable was eating 0 vs ≥ 1 fruit and vegetables per day, while for the second model, the dependent variable was eating ≥ 5 vs < 5 fruit and vegetables per day. The eight independent variables input into both models included: age, gender, neighbourhood deprivation (assessed by linking postcodes to the English Index of Multiple Deprivation [IMD] [11]), Body Mass Index (BMI), ethnicity, employment status, general health rating, and perceived importance of health. Before conducting regressions, missing data was imputed using the algorithm *MissForest*, which has been demonstrated to be effective at handling missing values in variables that have up to 30% missing information [12]. As sensitivity analyses, both models were redeveloped using only those participants with complete information.

## Results

Excluding *n* = 155 participants who did not respond to the relevant question, *n* = 822 (8.4%) participants reported that they never eat fruit and vegetables, while *n* = 1,461 (15.0%) reported that they generally eat ≥ 5 portions daily. Before conducting the regression models, *n* = 74 participants were excluded for two reasons: (1) missing data for too many variables (> 50%; *n* = 17); and (2) too few people identified as transgender (*n* = 57). This resulted in a final sample of *n* = 9840 for the regression models. Moreover, it was identified that the variable of age violated the assumption of linearity to the logit. As adding polynomials did not address this violation, age was treated as a categorical variable (i.e.,’18–34’,’35–64’, ‘65+’).

The results of the regression models (following missing data imputation) are shown in Table 2. Participants who never ate fruit and vegetables were significantly more likely to perceive healthy living as unimportant, identify as male, be younger than 65, be unemployed, or have a poor/very poor self-reported rating of overall general health (compared to having an excellent/good rating). Similarly, participants who ate < 5 portions of fruit and vegetables per day also perceived healthy living as unimportant, identified as male, were younger than 65, were unemployed, or had a moderate or poor/very poor rating of general health (compared to having an excellent/good rating). Neighbourhood deprivation was also identified as being associated with low fruit and vegetable consumption; however, both effect sizes were small.

**Table 2:** Factors associated with non-consumption and low consumption of fruit and vegetables (*n* = 9840)

Variable	Model 1 Eating 0 vs ≥ 1 Portions		Model 2 Eating ≥ 5 vs < 5 Portions	
	OR (95% CI)	p	OR (95% CI)	p
Age				
18–34	1		1	
35–64	0.98 (0.82–1.18)	0.826	1.05 (0.90–1.23)	0.517
65 +	0.47 (0.34–0.64)	< 0.001*	1.50 (1.24–1.83)	< 0.001*
Gender				
Male	1		1	
Female	0.55 (0.46–0.64)	< 0.001*	1.85 (1.64–2.08)	< 0.001*
IMD decile	0.91 (0.88–0.95)	< 0.001*	1.08 (1.05–1.10)	< 0.001*
BMI				
Healthy weight	1		1	
Underweight	1.15 (0.69–1.84)	0.580	1.02 (0.67–1.52)	0.908
Overweight	1.04 (0.86–1.26)	0.690	1.09 (0.94–1.26)	0.241
Obese	0.94 (0.78–1.12)	0.482	0.86 (0.75–1.00)	0.046^b^
Ethnicity				
White	1		1	
Non-white	0.97 (0.78–1.19)	0.752	0.88 (0.74–1.04)	0.149
Employment status				
Paid employment	1		1	
No paid employment	2.09 (1.60–2.79)	< 0.001*	0.70 (0.61–0.81)	< 0.001*
General health rating				
Excellent/good	1		1	
Moderate	1.22 (1.00–1.49)	0.047^b^	0.76 (0.66–0.87)	< 0.001*
Poor/very poor	2.28 (1.88–2.77)	< 0.001*	0.82 (0.70–0.95)	0.008*^a^
Perceived health imp				
No importance	1		1	
Some importance	0.31 (0.27–0.36)	< 0.001*	2.54 (2.05–3.19)	< 0.001*

*BMI* Body Mass Index, *IMD* Index of Multiple Deprivation, *OR* odds ratio

*Statistically significant when tested against a Bonferroni-adjusted alpha value of 0.025

^a^Not statistically sig. in sensitivity analysis (complete case data only; Supplementary Materials B)

^b^Statistically sig. in sensitivity analysis (complete case data only; Supplementary Materials B)

In sensitivity analyses in which only participants with complete information were included, similar findings were identified. However, two differences were observed: (1) having a moderate rating of general health was significantly associated with eating no portions per day; and (2) having obesity was associated with eating < 5 portions per day, while having a poor/very poor rating of general health was not (Supplementary Materials B).

## Discussion

Our findings highlight the low levels of fruit and vegetable consumption among people with SMI. Individuals who never consumed fruit and vegetables or ate < 5 portions per day were more likely to be male, younger than 65, unemployed, experience poorer general health, or perceive health as unimportant. These findings suggest that the characteristics of those individuals who never eat fruit or vegetables are similar to the characteristics of those who eat a limited amount (or less than recommended). Such individuals may require further assistance to help improve their dietary intake, especially considering that even modest increases in fruit and vegetable consumption can have notable beneficial effects on physical health [13, 14].

In terms of effective interventions developed for adults in the general population, behaviour-based interventions (i.e., those that apply behaviour theories or constructs, such as Theory of Planned Behaviour and self-efficacy), and interventions that consider environmental, educational, and multi-component factors, have been observed to increase fruit and vegetable intake [15-17]. Such developed interventions may provide guidance on how best to assist people with SMI to improve their dietary intake. In addition, other targeted interventions developed within the area of mental ill health may be instructive. For example, providing dietary advice and nutritional counselling support has been demonstrated to be effective at improving both diet quality and symptom severity for people with moderate-to-severe depression [18]. Further research should focus on developing similar interventions to help facilitate dietary improvement for adults with SMI. These require tailoring to address their more specific needs (e.g., potential impact of medication on food choice), potentially targeting factors identified in this study. For example, this study found that males consumed significantly lower levels of fruit and vegetables than females. Previous research suggests that interventions that apply the strategy of ‘choice architecture’ (i.e., influence eating behaviour through rearranging environmental structures) may be effective for improving dietary quality in men in the general population [19]. Such an approach may therefore be considered when developing tailored nutritional interventions for people with SMI.

A major strength of this study was that it utilised a large, nationally representative sample that was recruited from a range of areas and clinical contexts. However, the study may be limited by being conducted before the COVID-19 pandemic. Considering that inflation and food insecurity levels have substantially increased in recent times due to various factors (e.g., Russia-Ukraine war, climate crises, COVID-19 pandemic) [20], it is possible that fruit and vegetable consumption has further changed since participants were contacted. Moreover, the study was limited by its cross-sectional nature, which precluded causal effects and mechanisms from being investigated, and by the specific variables that were investigated in The CtG Health Study. Consequently, future research should examine other potentially relevant factors that were not investigated here (e.g., individuals’ food environments, experience of food insecurity [21], etc.), as well as other measures of diet quality (e.g., consumption of fast food, sweetened beverages, etc.). In conclusion, this study’s findings should help inform the design and delivery of interventions to improve dietary quality in people with SMI.

### Supplementary Information

Below is the link to the electronic supplementary material.

Supplementary file1 (PDF 1290 KB)

Supplementary file2 (DOCX 19 KB)

## Data Availability

The data that support the findings of this study are available from SG (simon.gilbody@york.ac.uk), upon reasonable request.
