# Supplementary material for: Factors associated with low fruit and vegetable consumption among people with severe mental ill health
Source: Soc Psychiatry Psychiatr Epidemiol. 2023 Jun 14;59(4):725–9. doi: 10.1007/s00127-023-02514-z (PMC10264868; doi:10.1007/s00127-023-02514-z)
Supplement: Supplementary file 1 — Supplementary file1 (PDF 1290 KB) [file 127_2023_2514_MOESM1_ESM.pdf]

16c. Would you like to cut down or quit smoking? ☐ Yes ☐ No ☐ Don't know

17. Have you ever tried an e-cigarette? ☐ Yes ☐ No **If you answered 'No' please go to question 18**

17a. Roughly how often in the last week have you used an e-cigarette?
☐ Every day ☐ Less than every day but at least once ☐ Never

18. What is your height? ______ metres OR ______ feet and inches

19. What is your weight? ______ kilograms OR ______ stone and pounds

Thank you for completing this questionnaire.

Consent form

May we contact you again about research that you may be interested in? ☐ Yes ☐ No

May we let your .... know that ☐ Yes ☐ No
you are taking part in this study? (They will not know your answers as they will remain confidential).

May we look at your health records to confirm your diagnosis and eligibility for research studies that you may be interested in taking part in? ☐ Yes ☐ No*

If I tick **yes** I understand that the information held and maintained by The Health and Social Care Information Centre and other central UK NHS bodies may be used to help contact me or provide information about my health status.

* You can still take part in the survey if you tick **no** to this question. If you tick no we will not look at your records.

Please fill in your forename, surname and address if different to those printed on the left:

First name: ....

Surname: ....

Address: ....

....

Please fill in your preferred contact details below:

Telephone: .... Mobile: ....

Email: ....

Signature: ....

Thank you for completing the Lifestyle Health and Wellbeing Survey.
Please put it in the prepaid envelope and post it to the researchers. No stamp is needed.

If you have any queries or would like any further information about this study please contact:
Emily Peckham on **01904 321 519** or email **emily.peckham@york.ac.uk**

Your .... has not given your name, personal or medical information to the University researchers, and the only information the researcher will receive will come from you if you decide to participate.

HWB *Lifestyle Health & Wellbeing survey*

UNIVERSITY of York

Health and Wellbeing Study

Closing the Gap

Welcome to the Lifestyle Health and Wellbeing Study

Please help us by filling in this questionnaire. Your answers will help us understand how we can improve the health of people with severe mental ill health. The questionnaire will take about 10 minutes to complete.

When you have completed the questionnaire, please return to the researchers at the University of York in the pre-paid envelope provided. You do not need a stamp.

CLOSING the GAP

General information

1. What is your date of birth?

☐☐ ☐☐ ☐☐☐☐
D D M M Y Y Y Y

2. Are you?

☐ Male ☐ Female ☐ Transgender ☐ Prefer not to say

3. How would you describe your ethnic background?

☐ White - British
☐ White - Irish
☐ Any other White background
☐ Mixed - White and Black Caribbean
☐ Mixed - White and Black African
☐ Mixed - White and Asian
☐ Any other mixed background
☐ Asian or Asian British - Indian
☐ Asian or Asian British - Pakistani
☐ Asian or Asian British - Bangladeshi
☐ Any other Asian background
☐ Black or Black British - Caribbean
☐ Black or Black British - African
☐ Chinese
☐ Other - please specify here:

....

4. How would you describe your employment status?

☐ Employed full-time (30+ hours per week)
☐ Employed part-time (<30 hours per week)
☐ Self-employed
☐ Retired
☐ Looking after family or home
☐ Student (full or part-time)
☐ Voluntary worker (paid or unpaid)
☐ Not employed but seeking work
☐ Not employed but not seeking work because of ill health
☐ Not employed, but not seeking work for some other reason
☐ Other - please specify

....

Your health

5. Do you have any physical or mental health condition lasting or expected to last 12 months or more?

☐ Yes ☐ No ☐ Don't know

6. How would you rate your general health in the last 12 months?

☐ Excellent ☐ Good ☐ Moderate ☐ Poor ☐ Very Poor

7. Does any health problem limit your activity?

☐ Yes ☐ No ☐ Don't know

Diet and fitness

8. How important is it that you maintain a healthy lifestyle?

☐ A top priority ☐ Moderately important ☐ I don't worry about it

9. In general, how often do you take part in any sport or physical activity?

☐ Every day
☐ Every other day
☐ At least once a week
☐ More than once a month but less than once a week
☐ Less than once a month
☐ Never

10. Would you like to take more exercise?

☐ Yes ☐ No ☐ Don't know

11. In general, how many portions of fruit and vegetables do you eat per day?

☐ I don't eat fruit or vegetables ☐ One ☐ Two ☐ Three ☐ Four ☐ Five or more

12. Would you like to change your diet or lose some weight?

☐ Yes ☐ No ☐ Don't know

Alcohol

13. How often do you have a drink containing alcohol?	Every day	Most days	Weekly	Monthly	Never

14. What is the most you drank in any one day in the last seven days?	0 units	1-2 units	3-4 units	5-6 units	7-9 units	10+ units
**15. How many units of alcohol do you drink on a typical day when you are drinking?**	0 units	1-2 units	3-4 units	5-6 units	7-9 units	10+ units

* **A unit of alcohol is equal to:** ½ a pint of ordinary beer, lager or cider; 1 single measure of spirits; 1 small glass of wine; or 1 measure of fortified wine.

Smoking

16. Do you smoke?

☐ Yes ☐ No I have never smoked ☐ No but I used to smoke

If you answered 'No' please go to question 17, overleaf

16a. What type of tobacco do you use?

☐ Packet cigarettes
☐ Cigars
☐ Water pipe/hookah/sheesha pipe
☐ Hand-rolled cigarettes
☐ Chewing tobacco
☐ Pipe
☐ Other, please specify

....

....

16b. How many cigarettes do you smoke per day?

☐ None ☐ 1-9 ☐ 10-19 ☐ 20 or more
