# Supplementary material for: Factors associated with low fruit and vegetable consumption among people with severe mental ill health
Source: Soc Psychiatry Psychiatr Epidemiol. 2023 Jun 14;59(4):725–9. doi: 10.1007/s00127-023-02514-z (PMC10264868; doi:10.1007/s00127-023-02514-z)
Supplement: Supplementary file 2 — Supplementary file2 (DOCX 19 KB) [file 127_2023_2514_MOESM2_ESM.docx]

| **Table B1.**  ***Factors Associated with Non-Consumption and Low-Consumption of Fruit and Vegetables (n=6067) – Complete Case Analysis*** | | | | |
| --- | --- | --- | --- | --- |
| Variable | Model 1  Eating 0 vs ≥1 Portions | | Model 2  Eating ≥5 vs <5 Portions | |
|  | OR (95% CI) | p | OR (95% CI) | p |
| Age |  |  |  |  |
| 18-34 | 1 |  | 1 |  |
| 35-64 | 0.84 (0.66-1.08) | 0.164 | 1.08 (0.89-1.32) | 0.434 |
| 65+ | 0.39 (0.25-0.59) | <0.001* | 1.66 (1.31-2.12) | <0.001* |
| Gender |  |  |  |  |
| Male | 1 |  | 1 |  |
| Female | 0.53 (0.42-0.66) | <0.001* | 1.84 (1.59-2.13) | <0.001* |
| IMD Decile | 0.91 (0.88-0.95) | <0.001* | 1.06 (1.03-1.08) | <0.001* |
| BMI |  |  |  |  |
| Healthy Weight | 1 |  | 1 |  |
| Underweight | 1.47 (0.76-2.67) | 0.228 | 0.79 (0.44-1.34) | 0.400 |
| Overweight | 1.01 (0.78-1.32) | 0.922 | 1.00 (0.84-1.19) | 0.988 |
| Obese | 0.92 (0.72-1.19) | 0.541 | 0.75 (0.63-0.90) | 0.002* |
| Ethnicity |  |  |  |  |
| White | 1 |  | 1 |  |
| Non-White | 0.83 (0.60-1.14) | 0.267 | 0.90 (0.71-1.14) | 0.404 |
| Employment Status |  |  |  |  |
| Paid Employment | 1 |  | 1 |  |
| No Paid Employment | 2.82 (1.93-4.27) | <0.001* | 0.71 (0.60-0.85) | <0.001* |
| General Health Rating |  |  |  |  |
| Excellent/Good | 1 |  | 1 |  |
| Moderate | 1.53 (1.17-2.02) | 0.002* | 0.74 (0.63-0.87) | <0.001* |
| Poor/Very Poor | 2.51 (1.91-3.32) | <0.001* | 0.82 (0.68-0.98) | 0.034 |
| Perceived Health Imp. |  |  |  |  |
| No Importance | 1 |  | 1 |  |
| Some Importance | 0.28 (0.23-0.35) | <0.001* | 2.71 (2.05-3.64) | <0.001* |
| *Statistically significant when tested against a Bonferroni-adjusted alpha value of 0.025.  Abbreviations: BMI, Body Mass Index; IMD, Index of Multiple Deprivation; OR, odds ratio. | | | | |
